# Bioactive Formulations in Agri-Food-Pharma: Source and Applications

**DOI:** 10.3390/bioengineering10020191

**Published:** 2023-02-02

**Authors:** Kandi Sridhar, Zeba Usmani, Minaxi Sharma

**Affiliations:** 1 Department of Food Technology, Karpagam Academy of Higher Education (Deemed to be University), Coimbatore 641021, India; 2 Department of Applied Biology, University of Science and Technology, Meghalaya 793101, India; 3 Haute Ecole Provinciale de Hainaut-Condorcet, 7800 Ath, Belgium

Bioactive compounds are the secondary metabolites produced by the plant cell through numerous metabolic pathways. Many epidemiological studies suggest that a diet rich in bioactive compounds can reduce the risk of degenerative diseases, such as diabetes, cancer, obesity, and cardiovascular complications by scavenging the free radicals generated in the body. Thus, there has been an increasing trend in the use of the natural bioactive compounds present in plant sources as antioxidants to scavenge free radicals. Recently, many agri-food pharma industries are exclusively searching for natural bioactive compounds and their formulations and approaches for the treatment of many degenerative diseases due to their low-cost production with no side effects. Hence, recent innovations in phytochemistry led to the extraction and identification of different novel bioactive compounds from a wide variety of plant sources, thereby application in food, cosmetic, and therapeutic industries. Nevertheless, appropriate extraction and identification strategies must be needed in order to recover the bioactive compounds from natural sources. These improvements in bioactive compound formulations make the process accessible and successfully implementable across various industries, particularly the agri-food pharma sector. Therefore, a group of researchers (Dr. Minaxi Sharma, Dr. Kandi Sridhar, and Dr. Zeba Usmani) with expertise in the area of bioactive compounds, plant-based foods, agri-food utilization, and plant-based drug development, organized the Special Issue on “Bioactive Formulations in Agri-Food-Pharma: Source and Applications, Volume II” to be published in *Bioengineering* (ISSN 2306-5354) under the section “Biochemical Engineering”. The Special Issue explored the most relevant and state-of-the-art high-quality research based on the following thematic areas:

- Recent developments in the production of natural compounds from various bioresources and their formulations as bioactives;
- Engineered approaches for enhanced biological properties;
- Potential industrial applications of new bioactive formulations through novel strategies;
- Identification of novel compounds and bioprocess tools to improve the quantity and quality of such products;
- Increasing knowledge on the bioactive formulations on regulation at the genomic and molecular levels.

This Special Issue provided a multidisciplinary view for the identification, extraction, and engineered application of bioactive compounds in agri-food pharma industries. Under this Special Issue topic, we invited renowned global researchers and received high-quality submissions. A total of 29 submissions were received for vigorous peer-review consideration, in which a total of 13 articles were accepted for final publication in this Special Issue. The total number of submissions indicated the importance of the research topic among researchers, policy makers, and other non-academic communities.

The Special Issue published a total of 13 scientific contributions comprising 9 research and 4 review articles which explored the bioactive compound sources and applications in the agri-food-pharma sector. These published articles are freely accessible from the moment of publication and part of a wider open movement (Copyright © 2022 or Copyright © 2023 by authors) under the terms and conditions of Creative Commons Attribution (CC BY) license to encourage the free exchange of knowledge and attract greater public engagement. The take-home message of the exciting articles published in this Special Issue are introduced below.

A study reported by Chuetor et al. [1] investigated the recyclability of ionic liquid, 1-ethyl-3-methylimidazolium acetate (EMIM-Ac) by optimization using rice straw. Under the optimized conditions, the authors studied the suitability of anti-solvent among water, acetone, methanol, and combinations of them. The study revealed that methanol was the best anti-solvent with the highest sugar yield. The combination of methanol and EMIM-Ac improved the recyclability of EMIM-Ac by up to five recycles. The recycled EMIM-Ac was used for the production of ethanol (89%), thereby confirming the potential of recycled ionic liquid in ethanol production at a low cost.

A study by Jo et al. [2] investigated various parts of *Prunus mume* for vasorelaxant effects, which could be related to the NO/sGC/cGMP vascular prostacyclin pathway. Similarly, another study by a Slovenian research group attempted to develop a plant-based drug against highly contagious severe acute respiratory syndrome coronavirus 2 (SARS-CoV-2) [3]. For this, authors used 70% aqueous ethanolic extract of the rhizome bark of Japanese knotweed and tested it on Vero-E6 cells (i.e., mimics the mechanism of the real virus–host interaction). A dosage of 50.8 µg/mL of plant extract significantly displayed the antiviral effect against SARS-CoV-2. This study concluded the use of Japanese knotweed rhizome bark extract in the formulation and development of food supplements for the complementary treatment of COVID-19 patients.

The results of an international collaboration among researchers from Egypt and Saudi Arabia provided insight regarding the production of cytokine (i.e., human interferon-α), of which regulates the immune system’s response to viral and bacterial infections from transgenic *Raphanus sativus* L. plants [4], indicating the *R. sativus* as a source for a biologically active compound that acts as an anticancer and antiviral drug. Similarly, another study by Wang et al. [5] investigated the neuroprotective effect of the Abelmoschus manihot flower extract obtained using ethanol, methanol, and supercritical fluid extraction against the cytotoxicity, oxidative stress, and inflammation in PC12 cells. A concentration of ethanolic and water extracts showed toxic effects on PC12 cells, while the supercritical extract caused 10% cell death with a more protective effect on the apoptosis of PC12 cells. The supercritical extracts up-regulated the expression of antioxidant enzymes, promoted the production of an intracellular antioxidant with reduced glutathione and reduced the reactive oxygen species generation in PC12 cells; this indicates the efficacy of supercritical extracts to repair the damage caused by oxidative stress and further delays the oxidative stress-induced neurodegenerative diseases. Likewise, Guo et al. [6] concluded the regulatory role of AdpA_1075 as a global regulator of morphological differentiation and secondary metabolism, promoting the biosynthesis of ansamitocin in *Actinosynnema pretiosum*.

Another research group from Portugal revealed the storage stability and in vitro bioaccessibility of the encapsulation of tomato extract and developed the yogurt enriched with the encapsulated tomato extract [7]. In vitro release studies showed a 63 and 13% release of encapsulated lycopene from the arabic gum and inulin particles in simulated gastric fluid, respectively. The microencapsulated lycopene added to yogurt showed the increased bioaccessibility during simulated gastrointestinal digestion compared to the microencapsulated lycopene alone, indicating the potential of encapsulation that can be used in the development of functional foods. By using the nanoencapsulation approach, Chen et al. [8] developed facile and novel lipid-based liposomes loaded with vitamins C and D3. This study indicated the high encapsulation efficiency for encapsulated vitamins with enhanced vitamin bioavailability. Another study by Alsubhi et al. [9] developed the polyphenol-enriched strawberry yogurt smoothie. In this study, authors extracted the polyphenols from pomegranate pomace and then added them to the strawberry yogurt smoothie. The addition of polyphenols enhanced the quality attributes and antioxidant activity of the strawberry yogurt smoothie, showing the potential of pomegranate pomace in the development of functional beverages.

A review by Das et al. [10] provided the overview on the cannabis (*Cannabis sativa* L.) bioactive compounds (i.e., cannabinoids), biosynthesis, post-harvest operations on the cannabinoid profile, drying treatments, and the effect of the different post-harvest operations on the cannabinoid yield. This review suggested the optimization of drying conditions, pre-treatment operations, and curing conditions in order to improve the extraction and identification of cannabinoids from cannabis. Since the bioactive compounds are sensitive to external and/or internal environments, many studies recommended the use of micro/nano encapsulation strategies, which were exemplified in a review by Puttasiddaiah et al. [11]. This review concluded the need of nanofabrication technologies for the encapsulation of bioactive compounds, thereby controlling the release of bioactive compounds and retaining the shelf-life of bioactive compounds to develop functional foods. Similarly, Rachitha et al. [12] reviewed the antihyperlipidemic activity of *Emblica officinalis* synergized with nanotechnological approaches, indicating the need of nanotechnological approaches in enhancing the antihyperlipidemic activity of *E. officinalis*. Another review by Bala et al. [13] provided novel approaches for the valorization of agro-waste into value-added bioproducts and bioactive compounds and explored the potential applications in the agri-food-pharma sector.

In conclusion, the advanced extraction, identification, and application of bioactive compounds reported in aforementioned studies can further advance the field and generate scientific knowledge. The novel strategies in the application of bioactive compounds are of interest to all stakeholders, encouraging them to look for new strategies to obtain bioactive compounds from natural sources and their application in agri-food, pharma, and chemical industries. More importantly, many of the studies featured in this article collection revealed the possibility in future research to identify novel natural bioactive sources from plants, which will be documented in future Special Issues.
